# The possible impact of an alcohol welfare surcharge on consumption of alcoholic beverages in Taiwan

**DOI:** 10.1186/1471-2458-13-810

**Published:** 2013-09-08

**Authors:** Chun-Yuan Yeh, Li-Ming Ho, Jie-Min Lee, Jhe-Yo Hwang

**Affiliations:** 1 Department of International Trade & Logistics, Overseas Chinese University, 100, Chiao-Kwang Rd., Taichung, Taiwan; 2 Department of Marine Leisure Management, National Kaohsiung Marine University, 142, Hai-Chuan Rd., Kaohsiung, Nan-Tzu, Taiwan; 3 Department of Shipping and Transportation Management, National Kaohsiung Marine University, 142, Hai-Chuan Rd. Nan-Tzu, Kaohsiung, Taiwan

**Keywords:** Alcohol prices, Welfare surcharge on alcoholic drinks, Price elasticity, Welfare surcharge revenues

## Abstract

**Background:**

The abuse of alcoholic beverages leads to numerous negative consequences in Taiwan, as around the world. Alcohol abuse not only contributes to cardiovascular disease, hypertension, diabetes and cancer, but it is also an underlying cause of many other serious problems, such as traffic accidents, lost productivity, and domestic violence. International leaders in health policy are increasingly using taxation as an effective tool with which to lower alcohol consumption. In this study, we assessed how consumption patterns in Taiwan would be affected by levying a welfare surcharge on alcoholic beverages of 20%, 40% or 60% in accordance with the current excise tax. We also assessed the medical savings Taiwan would experience if consumption of alcoholic beverages were to decrease and how much additional revenue a welfare surcharge would generate.

**Methods:**

We estimated the elasticity of four types of alcoholic beverages (beer, wine, whisky and brandy) using the Central Bureau of Statistics (CBS) Demand Model. Specifically, we estimated alcohol’s price elasticity by analyzing the sales prices and time statistics of these products from 1974 to 2009.

**Results:**

Alcoholic beverages in Taiwan have the following price elasticities: beer (−0.820), wine (−0.955), whisky (−0.587), brandy (−0.958). A welfare surcharge tax of 40% in accordance with the excise tax would decrease overall consumption of beer, wine, whisky and brandy between 16.24% and 16.42%. It would also generate New Taiwan Dollar (NT$) revenues of 5.782 billion to 5.993 billion. Savings in medical costs would range from NT$871.07 million to NT$897.46 million annually.

**Conclusions:**

A social and welfare surcharge of 40% on alcoholic beverages in Taiwan would successfully lower consumption rates, decrease medical costs, and generate revenue that could be used to educate consumers and further decrease consumption rates. Consequently, we strongly recommend that such a tax be imposed in Taiwan.

## Background

The abuse of alcoholic beverages increases risk for cardiovascular disease, gastrointestinal bleeding, cirrhosis of the liver, cancer, unintentional injuries, and violence [1]. Alcohol abuse also contributes to traffic accidents, lost productivity in the workplace, and domestic violence [2,3]. Consequently, decreasing consumption of alcoholic beverages has become a vital health policy worldwide.

In Taiwan, the medical costs associated with alcoholic fatty liver, alcoholic hepatitis, cirrhosis and other non-cancer diseases related to excessive drinking increased from NT$5.8 billion in 2006 to NT$6.6 billion in 2008 [4]. During the same time period, the tangible costs of alcohol consumption increased from NT$40 billion to NT$54 billion (about 0.4% of GDP) [5]. The percentage of traffic accidents caused by drunk drivers in Taiwan increased from 16.71% in 2004 to 26.93% in 2009. Fatalities due to alcohol-impaired driving increased from 453 deaths to 562 deaths during the same time period [6].

### Alcohol taxes

Countries around the world have begun to consider using taxation as a policy with which to curb alcohol consumption. For example, in 2010 the U.S. Task Force on Community Preventive Services (an independent body comprised of public health and prevention members appointed by the Director of the Centers for Disease Control and Prevention in Atlanta, Georgia) recommended that states raise their alcohol excise taxes to reduce excessive consumption and alcohol-related harm among the American population [7,8].

The government of Taiwan has put such recommendations into action. It currently levies an excise tax of NT$26 (US$0.78) on each liter of beer and of NT$2.5 (US$0.07) per liter of distilled liquor for each percent of alcohol content. Reprocessed alcoholic beverages are taxed NT$7 (US$0.21) for each percent of alcohol when the alcohol content is less than 20% by volume, and NT$185 (US$5.6) per liter when the alcohol content is over 20%.

In 2002, Taiwan levied a welfare surcharge on tobacco products that reduced cigarette consumption by 18% and saved the lives of between 28,125 and 56,250 people who otherwise would have died from tobacco-related illnesses. In addition, between NT$1.222 billion and NT$2.445 billion in annual health insurance expenses related to cigarettes were saved [9,10]. In contrast to tobacco, however, no similar surcharge has yet been levied on alcohol. Yet the effects of alcohol abuse on society—including traffic accidents and damage to the property and health of others—are just as serious (if not more so) than those of tobacco.

### The price elasticity of demand for alcoholic beverages

The impact of levying a health tax on alcoholic beverages depends on consumers’ response to price hikes. The price elasticity of demand is a measure of responsiveness of the quantity of a good or service demanded to the changes in its price. Elder et al. (2010) found that the median of the price elasticity of demand in all countries is −0.5 for beer, -0.64 for wine, and −0.79 for spirits. They also found that alcohol demand and prices are negatively correlated [11]. Wagenaar et al. (2009) found that the simple means of reported elasticities are −0.46 for beer, -0.69 for wine and −0.80 for spirits. They also found that price affects heavy consumption of alcohol significantly (mean reported elasticity = −0.28) [12]. Both Fogarty (2006) and Gallet (2007) found that alcohol price elasticities have decreased over time [13,14]. Research has also shown that beer is more inelastic than wine and spirits [11-14].

Babor et al. (2010) analyzed various strategies to determine which were most effective in reducing alcohol consumption. They found that price increases not only reduced consumption significantly, but that they also significantly reduced alcohol-related illnesses, such as cirrhosis of the liver [15]. Wagenaar et al. (2010) found that doubling the alcohol tax would reduce alcohol-related mortality by an average of 35% and deaths from traffic accidents by 11%. They further found that such measures would lower negative outcomes for alcohol related violence, sexually transmitted disease and crime [8]. Many researchers have concluded that alcohol taxes are the most cost-effective tools a government can use to reduce alcohol-related harm in developed and developing countries [16-18].

Lee et al. (2010) found that the price elasticity of alcoholic beverages in Taiwan is −0.77. They also found that a price increase of 10% would reduce consumption by 7.7% [19]. Wu (2010) found that if the current alcohol exercise tax in Taiwan were combined with a welfare surcharge of 40%, it would decrease consumption of pure alcohol by 7.3% and generate approximately NT$ 7.526 billion in revenue [20]. However, this study did not assess the cross effect of a welfare surcharge on specific types of alcoholic beverages. Because the relationship among beer, wine and spirits is complementary, the effect of levying a welfare surcharge on alcoholic beverages as one category is likely to substantially underestimate actual levels of consumption.

In economics, the cross elasticity of demand, or cross-price elasticity of demand, measures the responsiveness of the demand for a good to a change in the price of another good. A negative cross elasticity denotes two products that are complements of each other, and a positive cross elasticity denotes two products that are substitutes for each other. Studies have shown that beer and spirits are substitutes for each other in many countries, whereas beer and wine are complements for each other [21-24]. Few studies in Taiwan, however, have analyzed the price elasticity of beer, wine and spirits in detail [20].

### The goals of this study

To better understand the relationship between price and consumption of beer, wine and spirits in Taiwan, this study analyzed the elasticities of alcohol price/expenditure, the impact adding a tax surcharge to the excise tax would have on consumption, and the savings in medical costs that might result from lower consumption levels. The findings of this research will be particularly useful to public health officials who are considering using taxation as a tool with which to lower alcohol consumption.

### Alcohol consumption in Taiwan

**Figure 1:**
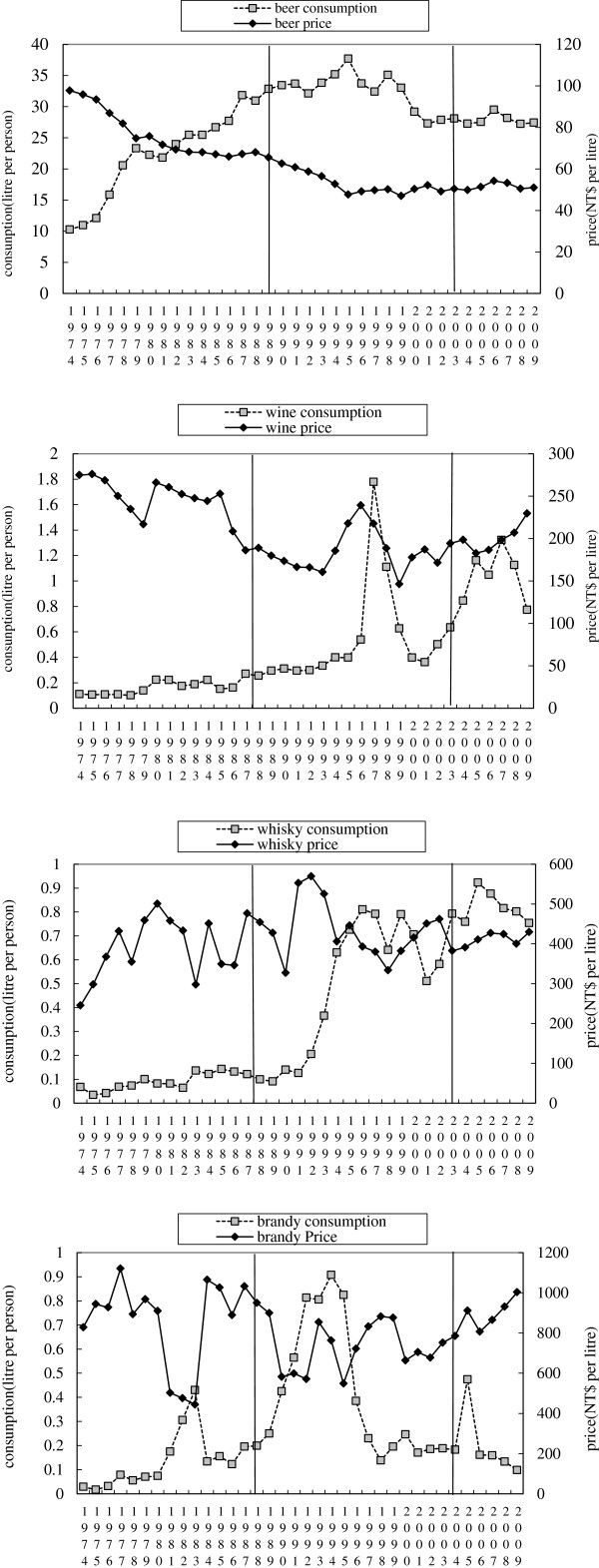
**The price and consumption of beer**, **wine**, **whisky and brandy in response to alcohol market opening in 1987 and tobacco health and welfare taxes levied in 2002.** Sources: Taiwan Tobacco and Wine Monopoly Bureau 1974–2000; National Treasury Agency 2001–09.

Consumption of beer in Taiwan reached a high of 37.69 liters per person in 1995; since then, it has gradually decreased. In the past decade, annual consumption of beer per capita has ranged from 27 to 28 liters. Figure 1 demonstrates that retail prices of beer in Taiwan have changed slightly every year; the long-term trend has been a gradual decrease in price. In the last decade, the retail price of beer per liter has remained around NT$50.

The amount of wine consumed in Taiwan has gradually increased since 1992. A large amount of wine was imported in 1997; simultaneously, wine consumption reached a high of 1.78 liters per person that year. After 1998, wine consumption gradually decreased due to falling demand and an economic slowdown. After Taiwan joined the WTO in 2002, however, wine consumption began to increase again. Over the past five years, the average amount of wine consumed per capita has been one liter. Although the overall long-term trend has been a gradual decrease in retail prices, wine prices increased slightly after 2002. In the past five years, the average retail price for wine has been approximately NT$200 per liter.

In contrast to beer and wine, consumption of whisky has increased noticeably in the last few years; however, consumption of brandy has decreased. Whisky imports into Taiwan have increased every year since 1991; they increased especially rapidly after 2002 and peaked in 2005. Today annual average consumption is 0.923 liters per capita per year. Since 2002, the retail price of whisky has been approximately NT$416 per liter.

Brandy was first imported into Taiwan in 1987; consumption rates increased gradually until hitting a peak in 1994, when an average of 0.908 liters were consumed per capita. After 1994, brandy consumption decreased rapidly; in the past five years, per capita consumption has averaged about 0.2 liters. Since 2005, the retail price of brandy has been approximately NT$903 per liter.

According to statistics from the Taiwanese Ministry of Finance, total consumption of alcoholic beverages was approximately 0.728 billion liters in 2011. Domestic production accounted for approximately 71.64% of this amount, whereas imports accounted for approximately 28.36%.

In regard to consumption of pure alcohol, beer (imported or domestic) comprised the largest share. In 2011, the consumption of beer reached 22.9 million liters; this translates to 37.99% of the total amount of pure alcohol consumed. Consumption of wine (76.7% of which was imported) totaled 3.27 million liters in 2011. This translates to approximately 5.42% of the total amount of pure alcohol consumed. Consumption of brandy totaled 1.52 million liters in 2011. This translates to approximately 2.52% of of the total. Consumption of whisky in 2011 was 8.72 million liter. This translates to approximately 14.47% of of the total. Altogether, approximately 36.41 liters of pure alcohol were consumed in Taiwan in 2011. About 60.4% of this amount came from beer, wine, whisky and brandy; about 39.6% came from other types of alcoholic beverages, such as other distilled liquors, other fermented liquors and compounded liquors.

## Methods

To evaluate the possible impact that a welfare surcharge of 20%, 40% or 60% would have on alcohol consumption rates, this study uses the Central Bureau of Statistics (CBS) Demand Model to estimate alcohol’s price elasticity.

### Data

The data used in this study come from the *Taiwan Tobacco and Wine Statistical Yearbook* published by the Taiwan Tobacco and Liquor Corporation, the tobacco and alcohol production and sales statistics of the National Treasury, and the import and export trade statistics of the Republic of China for the years 1974 to 2009 [25,26]. It is constituted from time series data that include total consumption and retail prices.

Consumption of alcoholic beverages was calculated as annual consumption per person, i.e., the total amount of beer, wine, whisky and brandy consumed annually in Taiwan divided by the total population over the age of 15. The population data were collected from the *Statistical Yearbook of Interior*, Republic of China, which is published by the Ministry of the Interior [27].

The average price of alcoholic beverages consisted of the weighted averages of the sale price of both imported alcohol and domestically produced alcohol. The price of domestically produced alcoholic beverages was mainly collected from the *Taiwan Tobacco and Wine Statistical Yearbook*[25]. The price of imported alcoholic beverages was based on import values divided by import volume. The prices for beer, wine, whisky and brandy consisted of wholesale prices that were deflated using the Consumer Price Index from 2001 as the base period.

Using the CBS demand model to evaluate the price elasticity of beer, wine, whisky and beer, we established the following settings [28]:

(1) $$w_{i}d\log x_{i}=\left(w_{i}+\theta_{i}\right)d\log Q+\sum_{j=1}\pi_{\mathrm{ij}}d\log p_{j}$$

In the above equation, w_i_ represents the average share of the budget of the i kind of alcoholic beverages (beer, wine, whisky and brandy), while *p*_i_ and x_i_ represent the price and amount of the i kind of alcoholic beverages; *d*log*p*_*j*_ represents the log change in the price for alcoholic product j; *d*log*Q* represents the amount index for Divisia for the change in real income. This can be written as *d*log*Q*= $\sum_{i}w_{i}d\log x_{i}$, where *d*log*x*_*i*_ represents the log change in the consumption level for alcoholic product i and θ_i_ and π_ij_ are fixed coefficients. The CBS demand function followed the economic assumptions of aggregation, symmetry and homogeneity restrictions.

We then sorted out the remaining endogenous variables for the CBS demand model [29]. These can be written as:

(2) $$dw_{i}=\theta_{i}d\log Q+\sum_{j=1}^{4}\left[\pi_{\mathrm{ij}}+w_{i}\left(\delta_{\mathrm{ij}}-w_{j}\right)\right]d\log p_{j}$$

Finally, we calculated the estimated elasticity for the uncompensated (Marshallian) price and expenditure. These are presented as ϵ_ij_ and *ϕ*_*i*_ respectively. The ϵ_ιj_ is the effect of an increase in the price of i on the quantity of j. The *ϕ*_*i*_ is the effect of an increase in the expenditure on the quantity of i. All can be derived from Equation (3) as follows:

(3) $$\epsilon_{\;\mathrm{ij}}=\frac{\pi_{\mathrm{ij}}+\theta_{i}w_{j}}{w_{i}}-w_{j}$$

(4) $$\phi_{i}=\frac{\theta_{i}}{w_{i}}+1$$

In this study, the CBS model constrained by homogeneity and symmetry conditions was implemented with Zellner’s Seemingly Unrelated Regression (SUR) procedure using the Time Series Processor (TSP) package version 4.5. Since the adding-up condition renders the system perturbation covariance matrix singular, estimation must take place after deletion of the brandy share equation, with estimates of the coefficients of the dropped equation retrieved from the adding-up constraints. The model also used estimated coefficient and average share of alcoholic beverages to calculate price and expenditure elasticity.

Following estimated price elasticities (see Table 1) and percentage change in price, we assumed that the percentage change in consumption was:

(5) pctdq=priceelasticity*pctdp

where pctdp is percentage change in price. We also defined the absolute decline in consumption as dq=pctdq*consumption. We subtracted the decline in consumption due to the price increase that occurs when a welfare surcharge is levied. The basic formula for dollars of tax revenue is:

(6) $$\begin{array}{l} \mathrm{tax}\;\mathrm{revenue}\;=\;\left(\mathrm{consumption}\;-\;\mathrm{dq}\right)\\ \;*\;\mathrm{welfare}\;\mathrm{surcharge} \end{array}$$

**Table 1:** Estimated uncompensated price and expenditure elasticity of alcoholic beverages

**Type of alcoholic products**	**Estimated expenditure elasticity**	**Estimated price elasticity**			
		**beer**	**wine**	**whisky**	**brandy**
beer	0.855** (21.987)	−0.820** (−19.327)	0.014 (1.662)	−0.044** (−3.904)	−0.002 (−0.153)
wine	2.207** (7.057)	−0.595 (−1.422)	−0.955** (−4.414)	−0.089 (−0.681)	−0.560** (−3.775)
whisky	1.051** (6.331)	−0.609** (−3.448)	0.0005 (0.014)	−0.587** (−5.854)	0.147* (1.950)
brandy	1.978** (7.034)	−0.931** (−3.194)	−0.142** (−3.619)	0.061 (0.823)	−0.958** (−7.258)

Note: 1. t values in brackets.

2 ** and * indicate 5% and 10% significance level for a two-tailed test.

The main method of imposing a welfare surcharge on alcoholic beverages is alcohol by volume (alc/vol) of alcohol or spirits. The average price and consumption levels of the four alcoholic beverages were calculated for the years 2005 to 2009. Our results show that the average price per liter during this time period was NT$52.04 for beer, NT$200.83 for wine, NT$ 903.36 for brandy, and NT$ 429.7 for whiskey. The average consumption per year was 546.51 million liters for beer, 20.41 million liters for wine, 4 million liters for brandy, and 16.02 million liters for whisky. The alcohol exercise tax was NT$26 per liter for beer, NT$100 per liter for brandy and whiskey (40% alc/vol), and NT$84 to NT$105 for wine (12% to 15% alc/vol).

## Results

### The elasticity of wine, beer, whisky and brandy

By estimating the uncompensated own price elasticity of the alcoholic beverages shown in Table 1, we found that the highest own price elasticity was −0.958 for brandy, -0.955 for wine, and −0.820 for beer. The own price elasticity for whisky was the lowest (−0.587). The fact that own price elasticity for wine, brandy and beer was higher than that for whisky means that consumers are more sensitive to price changes of wine, brandy and beer and less sensitive to the price changes of whisky. Consequently, imposing a welfare surcharge would decrease consumption in all four types of alcoholic beverages, but the decrease for whisky would not be as great.

The price elasticity of both wine and brandy was close to one, suggesting that price changes for these two products will not affect merchants’ revenues. The price elasticity of both beer and whisky was considerably less than one, which means that prices for these products have a positive relation to revenue: If the price of beer and whisky rises, merchants’ revenues will increase.

From the estimated uncompensated cross-price elasticity of alcoholic beverages, we found that beer and brandy—and beer and whisky—have complementary relationships. This means that when beer prices increase, not only beer consumption decreases, but also whisky and brandy consumption. On the other hand, we found that whisky and brandy have a weak substitution relationship. This means that when brandy prices increase, brandy consumption decreases while whisky consumption increases.

The expenditure elasticity of demand is a measure of the responsiveness of demand to changes in expenditure on similar goods. It reflects changes in quantity purchased and is also sensitive to changes in consumer expenditure. The estimated values of expenditure elasticity of all four types of alcohol reached statistical significance. As shown in Table 1, the highest expenditure elasticity was wine (2.207). Brandy was in the middle (1.978), and beer was the lowest (0.855).

### The effects of a 20%, 40%, or 60% welfare surcharge

This study found that if a welfare surcharge of 20% were levied, the price of beer would increase by 9.99% and decrease consumption by 8.19%. This is equivalent to 2,014,100 liters. It would have the greatest effect on brandy consumption, which would decrease by 9.3%. A 20% surcharge would increase the price of wine between 8.36% and 10.45% and decrease consumption by 7.98% to 9.98%. This is equivalent to 244,300 and 305,600 liters. It would increase the price of brandy by 2.21% and decrease consumption by 2.11%. This is equivalent to 36,400 liters. Finally, it would increase the price of whisky by 4.77% and decrease consumption by 2.8%. This is equivalent to 193,300 liters (See Table 2).

**Table 2:** **Analysis of the consumption effects of imposing a 20**%, **40**% **or 60**% **welfare surcharge on alcoholic products**

**Planning section welfare surcharge**	**Type of alcohol**	**Price changes(%)**	**Self**-**own and cross influence**	**Consumption changes(%)**	**Pure alcohol consumption (liters)**
Impose a 20% welfare surcharge in accordance with the excise tax amount	beer	9.99	beer	−8.19	−2,014,100
			wine	−5.95	−182,200
			whisky	−6.08	−418,800
			brandy	−9.30	−160,200
	wine	8.36~10.45	wine	−7.98~−9.98	−244,300~ −305,600
			brandy	−1.19~−1.49	−20,500~−25,600
			whisky	0.004~0.005	260~340
			beer	0.001	243
	whisky	4.77	whisky	−2.80	−193,300
			brandy	0.29	5,000
			beer	−0.21	−5,160,000
			wine	−0.42	−13,000
	brandy	2.21	brandy	−2.11	−36,400
			wine	−1.23	−37,800
			whisky	0.32	22,300
			beer	−0.005	−1,400
Impose a 40% welfare surcharge in accordance with the tax amount	beer	19.98	beer	−16.38	−4,028,300
			wine	−11.9	−364,400
			whisky	−12.16	−837,600
			brandy	−18.61	−320,400
	wine	16.73~20.91	wine	−15.97 ~ −19.97	−489,000~ −611,600
			brandy	−2.39~−2.98	−41,100~−51,300
			whisky	0.008~0.01	520~680
			beer	0.002~0.003	490~730
	whisky	9.55	whisky	−5.61	−386,600
			brandy	0.58	9,900
			beer	−0.42	−103,200
			wine	−0.85	−26,000
	brandy	4.42	brandy	−4.23	−72,900
			wine	−2.47	−22,700
			whisky	0.65	44,700
			beer	−0.01	−2,800
Impose a 60% welfare surcharge in accordance with the tax amount	beer	29.97	beer	−24.57	−6,042,300
			wine	−17.85	−546,600
			whisky	−18.24	−1,256,400
			brandy	−27.91	−480,600
	wine	25.095~31.36	wine	−23.95~−29.95	−733,400~−917,200
			brandy	−3.58~−4.47	−61,600~−76,900
			whisky	0.012~0.015	810
			beer	0.003~0.004	730~1,100
	whisky	14.32	whisky	−8.41	−579,900
			brandy	0.87	15,000
			beer	−0.63	−154,800
			wine	−1.27	−39,000
	brandy	6.63	brandy	−6.35	−109,200
			wine	−3.70	−113,400
			whisky	0.97	66,900
			beer	−0.02	−4,200

If a 40% surcharge were levied on beer, the price would increase by 19.98% and decrease beer consumption by 16.38%. This is equivalent to approximately 4,028,300 liters. Such an increase would also reduce the consumption of wine, whisky and brandy. It would have the greatest effect on brandy consumption, which would decrease by 18.61%. A 40% surcharge on wine would increase the price between 16.73% and 20.91% and decrease consumption by 15.97% to 19.97%. This is equivalent to between 489,000 and 611,600 liters.

A 40% tax on whisky would increase prices by 9.55% and decrease whisky consumption by 5.61%. This is equivalent to approximately 386,600 liters. A similar tax on brandy would increase prices by 4.42% and decrease brandy consumption by 4.23%. This is equivalent to approximately 72,900 liters.

If a welfare surcharge of 60% were levied, the price of beer would increase by 29.97% and decrease consumption by 24.57%. This is equivalent to 6,042,300 liters. Such an increase would also reduce the consumption of wine, whisky and brandy. It would have the greatest effect on brandy consumption, which would decrease by 27.91%.

A 20% welfare surcharge on all four products would reduce overall consumption of pure alcohol by 3.56 to 3.63 liters per person per year and generate approximately NT$3.239 to NT$3.318 billion in revenue. A 40% welfare surcharge would reduce overall consumption of pure alcohol by 6.93 to 7.14 million liters and generate approximately NT$5.782 to NT$5.933 billion in revenue. A 60% welfare surcharge on all four products would reduce overall consumption of pure alcohol by 10.11 to 10.34 liters and generate approximately NT$7.767 to NT$7.973 billion in revenue (See Table 3).

**Table 3:** **The effects on revenues of imposing a 20**%, **40**% **or 60**% **welfare surcharge on alcoholic products**

**Planning section welfare surcharge**	**Type of alcohol**	**Consumption (million liters)**	**Pure alcohol consumption (million liters)**	**Consumption changes (%)**	**Total revenues for a welfare surcharge on alcoholic products (NT$100 million)**
Impose a 20% welfare surcharge in accordance with the tax amount	beer	−47.61	−2.14	−8.71	25.94
	wine	−3.18~−3.59	−0.47~−0.53	−15.58~−17.58	2.82~3.61
	whisky	−1.73	−0.74	−10.83	2.93
	brandy	−0.49	−0.21	−12.30	0.70
	Total	−53.03~−53.43	−3.56~−3.63	−9.034	32.39~33.18
Impose a 40% welfare surcharge in accordance with the tax amount	beer	−91.86	−4.13	−16.80	47.28
	wine	−6.81~−7.69	−1.02~−1.15	−33.39~−37.68	4.27~5.71
	whisky	−3.47	−1.49	−21.67	5.02
	brandy	−0.68~−0.87	−0.29~−0.37	−17.02~−21.79	1.25~1.32
	Total	−95.37~ −96.43	−6.93~ −7.14	−16.24~−16.42	57.82~59.33
Impose a 60% welfare surcharge in accordance with the tax amount	beer	−128.99	−5.8	−23.60	65.13
	wine	−10.22~−11.54	−1.53~ −1.73	−50.1~−56.52	4.47~6.41
	whisky	−5.20~−5.21	−2.23~ −2.24	−32.51~−32.51	6.48
	brandy	−1.30~−1.34	−0.55~−0.57	−32.68~−33.45	1.59~.162
	Total	−145.73~−147.08	−10.11~−10.34	−24.82~−25.05	77.67~79.73

## Discussion

A welfare surcharge of 20%, 40% or 60% would increase the costs of alcoholic beverages, decrease consumption, and raise revenues. The resulting rise in costs, however, can be expected to generate resistance from both consumers and merchants (whose revenues will fall). A 20% surcharge will not raise enough revenue to offset the national health care costs related to overconsumption of alcoholic beverages. A 60% surcharge could be so onerous that it might be politically difficult to impose. Therefore, a 40% surcharge appears to achieve the best balance.

If a 40% welfare surcharge were imposed in conjunction with the current excise tax on all four alcoholic beverages, overall consumption would decrease by 16.24% to 16.42%. Beer and brandy, and beer and whisky, have a relatively high complementary relationship. This implies that demand for spirits falls when the price of beer increases. In other words, when it comes to deciding whether to drink beer, whisky or brandy, consumers’ decision-making process is parallel. If a welfare surcharge were imposed on beer, it would not only increase beer prices and decrease beer consumption, but it would also decrease consumption of brandy and whisky.

Furthermore, the welfare surcharge revenue would be between NT$5.782 billion and NT$ 5.933 billion. This is close to the medical expenditures on non-cancer diseases caused by excessive drinking. The combined medical costs of non-cancer diseases and traffic accidents caused by excessive drinking are approximately NT$7 billion annually [4]. In 2010, 55.69 million liters of pure alcohol were consumed in Taiwan [25]. This implies that the per liter medical cost of pure alcohol is NT$125.69. Consequently, a 40% welfare surcharge on alcoholic beverages would decrease consumption of pure alcohol by 6.93 to 7.14 million liters, saving between NT$871.07 million and NT$ 897.46 million in medical costs.

According to Taiwan’s Ministry of Finance, total revenue generated by the current alcohol tax is nearly NT$25 billion; clearly, the tax is insufficient to cover the tangible costs of drinking. Levying a 40% welfare surcharge on alcohol would go a long way toward compensating for such costs. From a public health and fiscal viewpoint, imposing a 40% welfare surcharge on alcoholic beverages would not only reduce alcohol consumption and increase government revenues, but it would also help to reduce the tremendous social costs of alcohol abuse.

Although a welfare surcharge on alcoholic beverages would have numerous benefits, it could also have some unintended consequences. For example, it could increase smuggling rates [30]. According to the High Level Group on Fraud in the Tobacco and Alcohol Sectors (1998), the EU lost an estimated €1.5 billion in tax revenue in 1996 as the result of smuggling; this is equivalent to approximately 8% of total revenue from alcohol taxes [31]. In 2001, the United Kingdom lost 4% of its alcohol tax revenue to smuggling [32].

Because the estimated price elasticity of all four products is less than one for each product, consumers would feel more pressure if a welfare surcharge were imposed than merchants would. However, merchants would also be affected by a welfare surcharge because revenues would decrease as consumption decreases. Therefore, authorities who levy such a tax should be prepared to experience significant resistance from merchants and other special interest groups.

## Conclusion

Levying a 40% welfare surcharge on alcoholic beverages would not only reduce overall levels of consumption, but it would also go a long way toward covering the current financial gap of the National Health Insurance and the financial expenditures for alcohol hazards control. Therefore, the study concludes that if the Taiwanese government were to levy such a tax, it would have a positive benefit on the entire country. For this reason, we strongly urge the government to adopt this measure.

If a welfare surcharge on alcoholic beverages is imposed, the government should enact an Alcohol Hazards Prevention Act to regulate the levy methods and use of collected surcharges. These additional revenues should be earmarked for education programs, rehabilitation centers and counseling that help individuals change their behavior toward alcohol.

## Competing interests

The author’s declare that they have no competing interests.

## Authors’ contributions

CYY and JML contributed to designing the study, conducting the statistical analysis, interpreting the empirical analysis and preparing the manuscript. LMH contributed to the conceptualization of the study and to data collection. JYH helped to prepare the manuscript and interpret the empirical analysis. All authors have read and approved the final manuscript.

## Pre-publication history

The pre-publication history for this paper can be accessed here:

http://www.biomedcentral.com/1471-2458/13/810/prepub
